# Supplementary material for: Cross-national disparities in healthcare workers’ perceptions: Examining fear of infection and confidence in the received COVID-19 vaccines amid emerging variants
Source: PLoS One. 2025 Dec 12;20(12):e0318788. doi: 10.1371/journal.pone.0318788 (PMC12700404; doi:10.1371/journal.pone.0318788)
Supplement: S1 File — (DOCX) [file pone.0318788.s002.docx]

**Table 1S. Healthcare workers' fears and perceptions towards SARS-CoV-2 emerging variants infection (n=5843)**

| **Emerging variants, SARS-CoV-2 fears, and perception** | **Strongly disagree** | | **Disagree** | | **Neutral** | | **Agree** | | **Strongly agree** | |
| --- | --- | --- | --- | --- | --- | --- | --- | --- | --- | --- |
|  | **No** | **%** | **No** | **%** | **No** | **%** | **No** | **%** | **No** | **%** |
| I am most afraid of the emerging variant of COVID-19. | 841 | 14.4% | 2092 | 35.8% | 1131 | 19.4% | 1128 | 19.3% | 651 | 11.1% |
| It makes me uncomfortable to think about the emerging variant of COVID-19. | 839 | 14.4% | 2239 | 38.3% | 1105 | 18.9% | 1197 | 20.5% | 463 | 7.9% |
| My hands become clammy when I think about the emerging variant of COVID-19. | 1402 | 24.0% | 2747 | 47.0% | 978 | 16.7% | 514 | 8.8% | 202 | 3.5% |
| I am afraid of losing my life because of the emerging variant of COVID-19. | 1100 | 18.8% | 2188 | 37.4% | 1080 | 18.5% | 848 | 14.5% | 627 | 10.7% |
| When watching news and stories about the emerging variant of COVID-19 on social media, I become nervous or anxious. | 1085 | 18.6% | 2313 | 39.6% | 1146 | 19.6% | 925 | 15.8% | 374 | 6.4% |
| I cannot sleep because I’m worrying about getting the emerging variant of COVID-19. | 1597 | 27.3% | 2703 | 46.3% | 879 | 15.0% | 449 | 7.7% | 215 | 3.7% |
| My heart races or palpitates when I think about getting the emerging variant of COVID-19 | 1730 | 29.6% | 2436 | 41.7% | 892 | 15.3% | 549 | 9.4% | 236 | 4.0% |

**Table 2S. Healthcare workers' confidence in the received COVID-19 vaccines (n=4251)**

| **Vaccine confidence** | **Strongly disagree** | | **Disagree** | | **Neutral** | | **Agree** | | **Strongly agree** | |
| --- | --- | --- | --- | --- | --- | --- | --- | --- | --- | --- |
|  | **No** | **%** | **No** | **%** | **No** | **%** | **No** | **%** | **No** | **%** |
| **I. Assessment of the confidence in vaccine effectiveness** | | | | | | | | | | |
| After receiving the vaccine, I might be infected with COVID-19 over the next 12 months. | 292 | 6.9% | 647 | 15.2% | 887 | 20.9% | 1677 | 39.4% | 748 | 17.6% |
| The COVID-19 Vaccine is likely to work with almost everyone | 203 | 4.8% | 673 | 15.8% | 910 | 21.4% | 1784 | 42.0% | 681 | 16.0% |
| The COVID-19 Vaccine would greatly strengthen (enhance) my immune system. | 169 | 4.0% | 410 | 9.6% | 971 | 22.8% | 1898 | 44.6% | 803 | 18.9% |
| Taking COVID-19 vaccine will give me complete freedom to get on with life just as before. | 210 | 4.9% | 571 | 13.4% | 1103 | 25.9% | 1627 | 38.3% | 740 | 17.4% |
| The speed of developing and testing the vaccine wouldn`t affect its effectiveness. | 237 | 5.6% | 671 | 15.8% | 1228 | 28.9% | 1455 | 34.2% | 660 | 15.5% |
| If individuals like me get the COVID-19 vaccine it will save many lives. | 133 | 3.1% | 251 | 5.9% | 922 | 21.7% | 1863 | 43.8% | 1082 | 25.5% |
| If many people do not get the vaccine this would be dangerous. | 144 | 3.4% | 322 | 7.6% | 895 | 21.1% | 1720 | 40.5% | 1170 | 27.5 |
| It is better to contract COVID-19 than to get the vaccination. | 705 | 16.6% | 1816 | 42.7% | 826 | 19.4% | 493 | 11.6% | 411 | 9.7% |
| **II. Assessment of the confidence in vaccine safety** | | | | | | | | | | |
| The speed of developing and testing the vaccine wouldn`t affect its safety. | 327 | 7.7% | 1031 | 24.3% | 1448 | 34.1% | 977 | 23.0% | 468 | 11.0% |
| The side effects for people of getting the COVID-19 vaccine will be significant | 49 | 1.2% | 1184 | 27.9% | 1379 | 32.4% | 1054 | 24.8% | 585 | 13.8% |
| Taking a new COVID-19 vaccine will make you feel like a (guinea pig). | 758 | 17.8% | 1271 | 29.9% | 1041 | 24.5% | 857 | 20.2% | 324 | 7.6% |
| The COVID-19-vaccine carries more risks than other vaccines. | 390 | 9.2% | 1241 | 29.2% | 1312 | 30.9% | 885 | 20.8% | 423 | 10.0% |
| **III. Assessment of the confidence in healthcare system** | | | | | | | | | | |
| The information I receive about the COVID-19-vaccine is trustful. | 188 | 4.4% | 489 | 11.5% | 1227 | 28.9% | 1751 | 41.2% | 596 | 14.0% |
| The information I receive about the COVID-19-vaccine from the vaccine program is reliable and trustworthy (duplicate). | 215 | 5.1% | 429 | 10.1% | 1228 | 28.9% | 1762 | 41.4% | 617 | 14.5% |
| The COVID-19-vaccine offered by the government program in my community is beneficial. | 201 | 4.7% | 443 | 10.4% | 1167 | 27.5% | 1704 | 40.1 | 736 | 17.3 |
| I trust the healthcare system that supports the vaccine. | 191 | 4.5% | 420 | 9.9% | 1118 | 26.3% | 1766 | 41.5% | 756 | 17.8% |
| I will go for the vaccines that are approved by national health system (duplicate). | 198 | 4.7% | 378 | 8.9% | 1035 | 24.3% | 1790 | 42.1% | 850 | 20.0% |


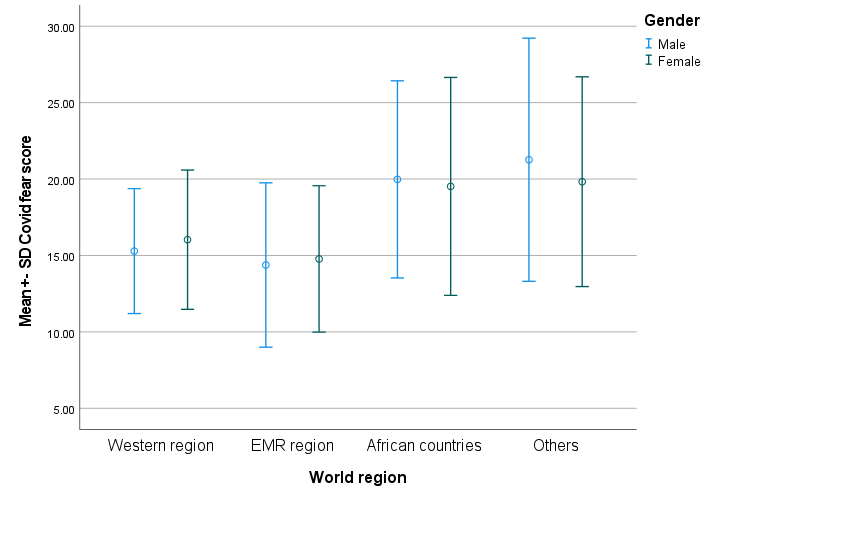


**Figure 1S. Overall SARS-Cov-2 emerging variants fear score by different respondents' regions and gender.** The error bar shows that the mean fear score was higher in females in the Western and EMR regions, but higher among males in the Africans and other regions.
