# Supplementary material for: Cross-national disparities in healthcare workers’ perceptions: Examining fear of infection and confidence in the received COVID-19 vaccines amid emerging variants
Source: PLoS One. 2025 Dec 12;20(12):e0318788. doi: 10.1371/journal.pone.0318788 (PMC12700404; doi:10.1371/journal.pone.0318788)
Supplement: S1 Questionnaire — (DOCX) [file pone.0318788.s003.docx]

1. Do you agree to participate in the study?
   - - 1. Yes, I agree
       2. No, I don’t agree
2. Age, please write a number in English.
3. Gender
   - - 1. Male
       2. Female
4. What is your Marital Status?
   - - 1. Married
       2. Single
       3. Widow
       4. Divorced
5. In what country do you live?
6. Place of residence
7. Urban/City
8. Rural
9. Desert region/ mountains
10. Others
11. Number of family members?
12. 1
13. 2
14. 3
15. 4
16. >=5
17. Level of income
18. Not enough and in debt
19. Not enough
20. Just enough
21. Enough and saving.

1. Field of study/practice in healthcare
2. Nursing
3. Medicine
4. Pharmacy
5. Health and rehabilitation sciences "Physiotherapy"
6. Dentistry
7. Administrative
8. Others
9. The highest education degree
10. Still in the internship year
11. Diploma
12. Bachelor science degree
13. Master's degree
14. Doctorate

*Suffered from chronic diseases?*

1. I don't suffer from any chronic diseases
   - - 1. Yes
       2. No
2. Hypertension
   - - 1. Yes
       2. No
3. Respiratory disease
   - - 1. Yes
       2. No
4. Immunological disease
   - - 1. Yes
       2. No
5. Diabetes
   - - 1. Yes
       2. No
6. Cardiac disease
   - - 1. Yes
       2. No
7. Renal disease
   - - 1. Yes
       2. No

1. Malignancy
   - - 1. Yes
       2. No
2. Other
   - - 1. Yes
       2. No

*Have you encountered any Mental Health Problems?*

1. I don't have any mental health problems.
   - - 1. Yes
       2. No
2. Stress Anxiety
   - - 1. Yes
       2. No
3. Sleep Disorders
   - - 1. Yes
       2. No
4. OCD
   - - 1. Yes
       2. No
5. Schizophrenia
   - - 1. Yes
       2. No
6. What is your smoking status?
   - - 1. Nonsmoker
       2. Current Smoker
       3. Ex-Smoker
7. Did any of your family member(s) get infected with COVID-19?
   - - 1. Yes
       2. No
       3. I don't know.
       4. Other:
8. Have you been infected with the Covid-19 virus?
   - - 1. Yes
       2. No
       3. Maybe

*History of previous COVID-19 infections*

1. Self-reported last covid infection
   - - 1. Less than 6 months
       2. From 6 to 12 months
       3. More than a Year
2. Needed hospitalization due to COVID-19 infection? *
   - - 1. Yes
       2. No
3. What was the severity of COVID-19 Symptoms?
   - - 1. Mild Symptoms
       2. Moderate Symptoms
       3. Severe Symptoms
       4. Needed Hospital Admission
       5. Needed ICU Admission
       6. Other:

*Vaccination History*

1. How many vaccinations did you receive? *
   - - 1. I didn't receive the vaccine
       2. Main Doses only
       3. One booster
       4. Two boosters
       5. More than two boosters
2. Were you obliged to take vaccination? *
   - - 1. Yes
       2. No

*If yes, what was the cause of the obligation?*

1. Work

Yes

No

1. Travel

Yes

No

1. Family Pressure

Yes

No

1. University Requirements

Yes

No

1. Peer Pressure

Yes

No

1. Entry to government facilities

Yes

No

1. Other

Yes

No

*Type of Vaccination?*

1. AstraZeneca

Yes

No

1. Pfizer

Yes

No

1. Sinopharm Sinovac

Yes

No

1. Johnson & Johnson

Yes

No

1. Moderna Sputnik

Yes

No

1. I don't know

Yes

No

*Did you face any side effects of the vaccination? **

1. No Side Effects

Yes

No

1. Pain at the site of injection

Yes

No

1. Fever

Yes

No

1. Flu--like Symptoms

Yes

No

1. Bone pain Allergy Headache Myalgia

Yes

No

1. Others

Yes

No

*Access confidence in COVID-19 Vaccines*

1. After receiving the vaccine, I might be infected with COVID-19 over the next 12 months.
   - - 1. Strongly Agree
       2. Agree
       3. Neutral
       4. Disagree
       5. Strongly Disagree

1. The COVID-19 Vaccine is likely to work with almost everyone
   - - 1. Strongly Agree
       2. Agree
       3. Neutral
       4. Disagree
       5. Strongly Disagree
2. The COVID-19 Vaccine would greatly strengthen (enhance) my immune system
   - - 1. Strongly Agree
       2. Agree
       3. Neutral
       4. Disagree
       5. Strongly Disagree
3. Taking COVID-19 vaccine will give me complete freedom to get on with life just as before.
   - - 1. Strongly Agree
       2. Agree
       3. Neutral
       4. Disagree
       5. Strongly Disagree
4. The speed of developing and testing the vaccine wouldn`t affect its effectiveness
   - - 1. Strongly Agree
       2. Agree
       3. Neutral
       4. Disagree
       5. Strongly Disagree
5. If individuals like me get the COVID-19 vaccine it will save many lives.
   - - 1. Strongly Agree
       2. Agree
       3. Neutral
       4. Disagree
       5. Strongly Disagree
6. If many people do not get the vaccine this would be dangerous
   - - 1. Strongly Agree
       2. Agree
       3. Neutral
       4. Disagree
       5. Strongly Disagree
7. It is better to contract COVID-19 than to get the vaccination
   - - 1. Strongly Agree
       2. Agree
       3. Neutral
       4. Disagree
       5. Strongly Disagree
8. The speed of developing and testing the vaccine wouldn`t affect its safety
   - - 1. Strongly Agree
       2. Agree
       3. Neutral
       4. Disagree
       5. Strongly Disagree
9. The side effects for people of getting the COVID-19 vaccine will be significant
   - - 1. Strongly Agree
       2. Agree
       3. Neutral
       4. Disagree
       5. Strongly Disagree
10. Taking a new COVID-19 vaccine will make you feel like a (guinea pig).
    - - 1. Strongly Agree
        2. Agree
        3. Neutral
        4. Disagree
        5. Strongly Disagree
11. The COVID-19-vaccine carries more risks than other vaccines
    - - 1. Strongly Agree
        2. Agree
        3. Neutral
        4. Disagree
        5. Strongly Disagree
12. The information I receive about the COVID-19-vaccine is trustful
    - - 1. Strongly Agree
        2. Agree
        3. Neutral
        4. Disagree
        5. Strongly Disagree
13. The information I receive about the COVID-19-vaccine from the vaccine programis reliable and trustworthy.
    - - 1. Strongly Agree
        2. Agree
        3. Neutral
        4. Disagree
        5. Strongly Disagree
14. The COVID-19-vaccine offered by the government program in my community is beneficial.
    - - 1. Strongly Agree
        2. Agree
        3. Neutral
        4. Disagree
        5. Strongly Disagree
15. I trust the healthcare system that supports the vaccine
    - - 1. Strongly Agree
        2. Agree
        3. Neutral
        4. Disagree
        5. Strongly Disagree
16. I will go for the vaccines that are approved by national health system
    - - 1. Strongly Agree
        2. Agree
        3. Neutral
        4. Disagree
        5. Strongly Disagree

*Fear of COVID-19*

1. I am most afraid of coronavirus-19.
   - - 1. Strongly Agree
       2. Agree
       3. Neutral
       4. Disagree
       5. Strongly Disagree
2. It makes me uncomfortable to think about coronavirus-19. *
   - - 1. Strongly Agree
       2. Agree
       3. Neutral
       4. Disagree
       5. Strongly Disagree
3. My hands become clammy when I think about coronavirus-19. *
   - - 1. Strongly Agree
       2. Agree
       3. Neutral
       4. Disagree
       5. Strongly Disagree
4. I am afraid of losing my life because of coronavirus-19. *
   - - 1. Strongly Agree
       2. Agree
       3. Neutral
       4. Disagree
       5. Strongly Disagree
5. When watching news and stories about coronavirus-19 on social media, I become *
   - - 1. Strongly Agree
       2. Agree
       3. Neutral
       4. Disagree
       5. Strongly Disagree
6. I cannot sleep because I’m worrying about getting coronavirus-19.
   - - 1. Strongly Agree
       2. Agree
       3. Neutral
       4. Disagree
       5. Strongly Disagree
7. My heart races or palpitates when I think about getting coronavirus-19 *
   - - 1. Strongly Agree
       2. Agree
       3. Neutral
       4. Disagree
       5. Strongly Disagree
