# Supplementary material for: Cross-national disparities in healthcare workers’ perceptions: Examining fear of infection and confidence in the received COVID-19 vaccines amid emerging variants
Source: PLoS One. 2025 Dec 12;20(12):e0318788. doi: 10.1371/journal.pone.0318788 (PMC12700404; doi:10.1371/journal.pone.0318788)
Supplement: Attachment — Submitted filename: Response to Reviewers.docx [file pone.0318788.s007.docx]

**Dear Editor-in-Chief**

***Journal of PLOS ONE***

# Manuscript No: (PONE-D-24-28390)

**Title: “Cross-National Disparities in Healthcare Workers' Perceptions: Examining Fear of Infection and Confidence in COVID-19 Vaccines in the Context of Emerging Variants”**

**Dear esteemed reviewers**, thank you for your revision request and your sincere efforts, which helped us improve our manuscript's quality. Per your suggestions, we have carefully revised the manuscript and rechecked the main text point by point.

| **Editor’s comments:** | **Author’s response** |
| --- | --- |
| **Editorial comments** | |
| 1. Please provide additional details regarding participant consent. In the ethics statement in the Methods and online submission information, please ensure that you have specified (1) whether consent was informed and what type you obtained (for instance, written or verbal, and if verbal, how it was documented and witnessed). | Response: We added the following details to the Ethical Considerations and Approval section of the Method. All participants were informed that their participation was voluntary and informed written consent was obtained by answering the first question before starting the survey (“to agree” or “not to agree”) to participate in the study. |
| 2. If your study included minors, state whether you obtained consent from parents or guardians. If the need for consent was waived by the ethics committee, please include this information. | Thank you for raising this issue. In this study, we included adults aged 18 years or above. In this research, we included healthcare workers to assess their fear of new variants of COVID-19 and their confidence in the received vaccine. |
| 3. If you are reporting a retrospective study of medical records or archived samples, please ensure that you have discussed whether all data were fully anonymized before you accessed them and/or whether the IRB or ethics committee waived the requirement for informed consent. | Response: Not applicable. |
| 4. If patients provided informed written consent to have data from their medical records used in research, please include this information. | Response: Non-applicable |
| 5. Once you have amended this/these statement(s) in the Methods section of the manuscript, please add the same text to the “Ethics Statement” field of the submission form (via “Edit Submission”). | *Done as requested* |
| 6. Please include a complete copy of PLOS’ questionnaire on inclusivity in global research in your revised manuscript. | I have attached a copy of PLOS’ questionnaire on inclusivity in global research |
| 7. Please upload a completed version of your questionnaire as Supporting Information when you resubmit your manuscript. | A copy of the questionnaire was uploaded as a supplementary file. |
| 8. Providing interested researchers with a durable point of contact ensures data will be accessible even if an author changes email addresses, institutions, or becomes unavailable to answer requests. Before we proceed with your manuscript, please also provide non-author contact information (phone/email/hyperlink) for a data access committee, ethics committee, or other institutional body to which data requests may be sent. If no institutional body is available to respond to requests for your minimal data, please consider if there any institutional representatives who did not collaborate in the study, and are not listed as authors on the manuscript, who would be able to hold the data and respond to external requests for data access? If so, please provide their contact information (i.e., email address). Please also provide details on how you will ensure persistent or long-term data storage and availability. | We uploaded the data for repository and will be available for anyone.  <https://osf.io/kmnsc/files/osfstorage?view_only=> |
| 9. PLOS requires an ORCID iD for the corresponding author in Editorial Manager on papers submitted after December 6th, 2016. Please ensure that you have an ORCID iD and that it is validated in the Editorial Manager | I have attached my ORCID |
| 10. Please amend your list of authors on the manuscript to ensure that each author is linked to an affiliation. Authors’ affiliations should reflect the institution where the work was done (if authors moved subsequently, you can also list the new affiliation stating “current affiliation:….” as necessary). | Checked |
| 11. Your ethics statement should only appear in the Methods section of your manuscript. If your ethics statement is written in any section besides the Methods, please delete it from any other sections. | Done as requested |

| **Reviewers' comments:** | | **Author’s response** |
| --- | --- | --- |
| **Reviewer #1:** | | |
| Kindly define the outcome terms in the methods section - for example normal fear/extreme fear. What do they mean? What were their cut-offs? How did you come to that | Thank you for your valuable comment. We would like to clarify that the definitions of "normal fear" and "extreme fear" were included in the original manuscript under the "Data Collection Tools" section. Specifically, fear levels were assessed using the Fear of COVID-19 Scale (FCS), a 7-item scale with a score range from 7 to 35, where higher scores indicate greater levels of fear. As per previously published literature [reference 34], a score above 17.5 was considered indicative of "extreme fear," while scores below this threshold were classified as "normal fear."  For further clarity, the methods go as follows:  “The best cutoff for FCS was at 17.5 with a sensitivity of 55.1% and specificity of 49.6%, AUC = 0.520 was statistically significant (p-value < 0.001), a confidence interval of 95% CI = [0.502, 0.538. So, those with a total score above 17.5 were considered as cases with extreme fear, while those below 17.5 were known as under normal fear of COVID-19 [1]." To further enhance clarity, we have now emphasized these definitions in the Introduction of the revised manuscript. In the Introduction, we added:  “According to The Diagnostic and Statistical Manual of Mental Disorders, Fifth Edition, fear is defined as an emotional response to a real or perceived imminent threat[2]. It is a crucial survival emotion, triggering adaptive defense mechanisms in response to danger, and may contribute to the onset of mental health disorders or worsen conditions like generalized anxiety, depression, and suicide[3].” This theoretical framework supports our use of the FCS in assessing COVID-19-related fear and the cut-off points used. | |
| What is good confidence/intermediate/poor confidence | Thank you for your question. In the study, we assessed confidence in COVID-19 vaccines using the **ARAB-VAX-CONF** tool, which measures confidence across three domains: vaccine effectiveness, vaccine safety, and trust in the healthcare system. Each domain was scored on a 5-point Likert scale, with responses ranging from 1 (strongly disagree) to 5 (strongly agree).  We categorized confidence into three levels based on the participants' composite mean scores across these items  Here is part of the method for more clarification “Overall confidence was obtained through calculating the composite mean score for the different confidence items discrete score (ranged from 1 to 5). Participants with a mean composite score < 2.5 were considered with poor confidence, (2.5-3.49) were considered with intermediate confidence, and those with a mean composite score of (3.5-5) were considered with good confidence[4].”  These cut-offs were determined based on the scale's validation and prior literature that used similar scoring systems to assess vaccine confidence [reference 46].  **Changes in the manuscript:** To ensure clarity, we have revised the **Methods (Data Collection Tools)** section to further explain these cut-offs. We added the following text: “Confidence in COVID-19 vaccines was assessed using the ARAB-VAX-CONF tool. Participants were classified into three categories based on their composite mean scores: good confidence (3.5–5), intermediate confidence (2.5–3.49), and poor confidence (<2.5). These thresholds were determined based on the validation of the tool and previous studies on vaccine confidence [reference 46]. | |
| **Reviewer #2:** | | |
| 12. The introduction should provide more background on the emergence of new COVID-19 variants and their potential impact on healthcare workers' perceptions and behaviors. In my opinion, this is crucial for understanding the study's relevance. | | Thank you for your feedback. We have expanded the introduction to include a more detailed discussion of SARS-CoV-2 variants and their impact on healthcare workers' perceptions and behaviors.  The revised introduction now includes the following additions:   - A detailed classification of SARS-CoV-2 variants and their implications [4, 5]. - Specific mention of the most recent Omicron variant and its impact on public health [6]. - An explanation of how these variants may exacerbate healthcare workers' perceptions and behaviors from the literature [12-14].   We added these two new parts as follows:  *“The World Health Organization (WHO) and the Centers for Disease Control and Prevention (CDC) have identified several SARS-CoV-2 variants, categorized into variants of concern, variants of interest, and variants of high consequence. These variants include Alpha, Beta, Gamma, and Delta, with the most recent addition being the Omicron variant [4, 5].”*  *“Following the emergence of the Omicron variant, concerns were raised about the vaccine's effectiveness against this strain. A study involving 1,285 HCWs reported that HCWs aged 55 or older found a significant perception of vaccine ineffectiveness in controlling the spread of Omicron [12]. Similarly, in a study conducted after the Delta variant outbreak, over 44% of HCWs participants declined a booster dose, and one-third preferred a new mRNA vaccine specifically developed for the new variant [13]. These concerns affected both vaccination rates and the mental health of HCWs. Allawi et al. reported that 66% of HCWs experienced varying degrees of anxiety and depression, with a vaccination rate of only 21.3%. This low uptake was primarily attributed to a lack of confidence in the vaccines' ability to prevent infection [14]”* |
| 13. A brief review of previous studies on healthcare workers' attitudes towards COVID-19 vaccines and their fears related to infection would be beneficial, as this would help establish the current state of knowledge and identify gaps this study aims to fill. Additionally, the authors could strengthen the rationale for their study by explaining why understanding healthcare workers' perceptions is particularly important in the context of emerging variants. | | Thank you for your insightful feedback. We have addressed your suggestion by incorporating a brief review of previous studies on healthcare workers' (HCWs) attitudes towards COVID-19 vaccines and their fears related to infection.  In the revised introduction, we have included the following updates:  **Review of Previous Studies**:   - An explanation of how these variants may exacerbate healthcare workers' perceptions and behaviors from the literature [12-14]. - The definitions of fear and its impact on mental health [3]. - Reports from the literature regarding fears related to infection among HWCs and the importance of addressing these issues for preventing outbreaks [34-37]   Here are new parts added in the introduction:  *“Following the emergence of the Omicron variant, concerns were raised about the vaccine's effectiveness against this strain. A study involving 1,285 HCWs reported that HCWs aged 55 or older found a significant perception of vaccine ineffectiveness in controlling the spread of Omicron [12]. Similarly, in a study conducted after the Delta variant outbreak, over 44% of HCWs participants declined a booster dose, and one-third preferred a new mRNA vaccine specifically developed for the new variant [13]. These concerns affected both vaccination rates and the mental health of HCWs. Alalawi et al. reported that 66% of HCWs experienced varying degrees of anxiety and depression, with a vaccination rate of only 21.3%. This low uptake was primarily attributed to a lack of confidence in the vaccines' ability to prevent infection [14]”*  *“According to The Diagnostic and Statistical Manual of Mental Disorders, Fifth Edition, fear is defined as an emotional response to a real or perceived imminent threat [2]. It is a crucial survival emotion, triggering adaptive defense mechanisms in response to danger, and may contribute to the onset of mental health disorders or worsen conditions like generalized anxiety, depression, and suicide[3].”*  *“A study on 2,336 HWCs reported an increasing rate of severe CoViD-19 fear from 9% to 15%[5].Besides impacting their well-being, fear also has a detrimental impact on their occupational outcomes. This was reported in other studies in different regions as well[6, 7] Addressing HWC's fear is crucial for the optimization of their professional performance in preventing outbreaks and as a resource for patient and public health education and awareness[7, 8] ”* |
| 14. The authors may consider incorporating a brief discussion on the potential neural substrates underlying fear responses and vaccine confidence among healthcare workers. Recent neuroimaging studies have identified brain regions involved in processing fear and anxiety, such as the amygdala, insula, and prefrontal cortex, which could be relevant to understanding the neurobiological basis of COVID-19 related fears. Additionally, exploring the neural correlates of decision-making processes related to vaccine acceptance could provide valuable insights. Including this perspective could strengthen the paper's theoretical framework and suggest directions for future research combining psychological and neuroscientific approaches to vaccine hesitancy among healthcare professionals [1-3]. | | Thank you for this valuable suggestion. In response to this point, we have incorporated a new paragraph into the Discussion section to address the potential neural substrates underlying fear responses and vaccine confidence among healthcare workers. The added paragraph is as follows:  "The neural substrates underlying fear responses and vaccine confidence among HCWs may help understand the psychological and neurobiological factors influencing vaccine acceptance. Key brain regions, such as the amygdala, insula, and prefrontal cortex, play important roles in processing fear, risk perception, and decision-making (Broche-Pérez et al., 2016). This complexity underscores the importance of integrating psychological and neuroscientific perspectives in future research to address vaccine hesitancy among healthcare professionals." |
| 15. The manuscript lacks details on how participants were recruited across different regions. More information is needed on the sampling method and how representativeness was ensured. | | Thank you for your comment. we have revised the Methods section to provide additional details on how participants were recruited across different regions and how we aimed to ensure representativeness.  Specifically, we used convenience and snowball sampling to recruit healthcare workers (HCWs) from the four world regions: Eastern Mediterranean Region (EMR), African Region (AFR), Western countries, and other world regions (Eastern Asia and Latin America).  *The co-author (AG) was responsible for recruiting collaborators from a proportionate number of countries representing the four regions of the world via the Global Researcher Club—an international, voluntary, and non-profit scientific research network.*  Through this network, collaborators from a proportionate number of countries within each region were contacted to participate in and further disseminate the survey among HCWs in their respective countries. This strategy helped ensure broader geographic representation. |
| 16. While they mention both online surveys and face-to-face interviews were used, authors have provided no explanation of how face-to-face interviews were conducted safely during the pandemic. The process for distributing the online survey is also not described: authors should provide more details on this. | | Thank you for your question. The online survey was created by Google Forms and distributed through social media platforms (Facebook and Twitter), and sent via (email, WhatsApp, and Telegram). The collaborators were responsible for sharing the survey to target population at their country/region. The study was conducted between 1^st^ November to 5^th^ December 2023. As of May 2023, the WHO ended the Public Health Emergency of International Concern status for COVID-19 which facilitated the face-to-face data collection[9].  The face-to-face interview was conducted through a printed hard copy of the study questionnaire delivered to the target population and then collected back after filling out the questionnaire. |
| 17. The methods section does not describe any statistical analysis techniques used to analyze the data. This is a significant omission. | | Thank you for your valuable comments. we  have revised the Methods section to provide additional details on statistical analysis techniques used during analysis. |
| 18. There's no mention of statistical tests performed or p-values to indicate the significance of the findings. | | Thank you for your comments. we have revised the Methods section to provide additional details on statistical tests performed used during analysis. or p-values indicating the significance of the findings |
| 19. The results don't address potential confounding factors that could influence fear levels or vaccine confidence among healthcare workers. | | Thank you for raising this important point. We agree that addressing potential confounding factors is crucial for the interpretation of our findings. To this end, we conducted a multivariable analysis using stepwise logistic regression models to control for potential confounders and better understand the determinants of healthcare workers' fear of COVID-19 infection and their confidence in the COVID-19 vaccines.  In our analysis, we identified several key factors associated with vaccine confidence and fear levels:  **Confidence in the COVID-19:** Table 9. Multiple stepwise logistic regression model for determinants of health care workers' good confidence regarding COVID-19 vaccine.  Higher confidence was reported among old age participants [AOR: 1.10; 95% CI (1.01-1.12); p-value= 0.001], males[AOR: 1.28; 95% CI (1.13-1.46); p-value= 0.001], those with chronic [AOR: 1.22; 95% CI (1.06-1.41); p-value= 0.006] and mental disorders [AOR: 1.10; 95% CI (1.01-1.12); p-value= 0.001], high family number[AOR: 1.65; 95% CI (1.42-1.91); p-value= 0.001], and personal [AOR: 1.18; 95% CI (1.06-1.31); p-value= 0.002] or family history of covid infection [AOR: 1.25; 95% CI (1.11-1.40); p-value= 0.001]. Experiencing side effects was associated with a low confidence regarding the vaccine [AOR: 0.63; 95% CI (0.55-0.72); p-value= 0.001].  **Fear of COVID-19**: Table 7 shows factors as the most significant determinants associated with fear of COVID-19 infection. Extreme fear was reported among African countries (64.6%), divorced / widow (48.9%), those had four family members (41.6%), without enough monthly income and in debit (54.4%), nursing (50.3%), and didn’t suffer from any chronic disease (485%). |
| 20. The discussion would benefit from comparing these findings to other relevant studies on healthcare worker vaccine confidence and fear levels during the pandemic | | We incorporate additional studies to compare our findings with existing literature on healthcare workers' fear and vaccine confidence levels, as suggested by the reviewer. The new sections are marked with yellow highlights. |
| 21. While regional differences are mentioned, there's minimal exploration of potential reasons behind these disparities. I would suggest expanding on this point. | | Potential reasons behind these disparities were discussed and marked with yellow highlights |
| 22. The conclusions could be strengthened with more specific recommendations for practice and policy based on the findings. | | Thank you for your suggestion. we have revised the conclusions to include more specific, actionable recommendations based on the study findings. The updated conclusions now emphasize tailored strategies for addressing both fear of infection and vaccine confidence among healthcare workers (HCWs), with a focus on regional and demographic differences.  The new refined recommendations in the manuscript as:- *“Future interventions and policies should be tailored to address regional and demographic variations in fear levels and vaccine confidence, promoting widespread vaccine acceptance among HCWs. Additionally, fear screening tools should be employed to identify HCWs experiencing heightened fear, particularly women, to enable more targeted interventions. Personalized psychological support and programs that encourage open communication should also be implemented to address these concerns effectively.”* |

References:
1. https://doi.org/10.1038/s41398-020-01150-4
2. https://doi.org/10.1111/acps.13602
3. DOI: 10.3390/biomedicines12051083

1.	Mohsen, F., et al., *Fear among Syrians: A Proposed Cutoff Score for the Arabic Fear of COVID-19 Scale.* PloS one, 2022. **17**(3): p. e0264257.

2.	Edition, F., *Diagnostic and statistical manual of mental disorders.* Am Psychiatric Assoc, 2013. **21**(21): p. 591-643.

3.	Gunnell, D., et al., *Suicide risk and prevention during the COVID-19 pandemic.* The Lancet Psychiatry, 2020. **7**(6): p. 468-471.

4.	Abuyadek, R., et al., *Development and validation of Tool for Assessment of post vaccination confidence in COVID 19 Vaccines.* 2023.

5.	Moretti, M., et al., *Fear for CoViD-19 and reluctance to work among health care workers during the epidemic, a prospective monocentric cohort study.* American Journal of Infection Control, 2022. **50**(3): p. 312-318.

6.	Mendes, F.R., et al., *Fear of COVID-19 among professional caregivers of the elderly in Central Alentejo, Portugal.* Scientific Reports, 2024. **14**(1): p. 3131.

7.	Troisi, A., et al., *Fear of COVID-19 among Healthcare Workers: The Role of Neuroticism and Fearful Attachment.* J Clin Med, 2021. **10**(19).

8.	Kinman, G., K. Teoh, and A. Harriss, *Supporting the well-being of healthcare workers during and after COVID-19.* Occup Med (Lond), 2020. **70**(5): p. 294-296.

9.	Sarker, R., et al., *The WHO has declared the end of pandemic phase of COVID-19: Way to come back in the normal life.* Health Sci Rep, 2023. **6**(9): p. e1544.
