# Supplementary material for: Cross-national disparities in healthcare workers’ perceptions: Examining fear of infection and confidence in the received COVID-19 vaccines amid emerging variants
Source: PLoS One. 2025 Dec 12;20(12):e0318788. doi: 10.1371/journal.pone.0318788 (PMC12700404; doi:10.1371/journal.pone.0318788)
Supplement: Attachment — Submitted filename: RTR_auresp_2.docx [file pone.0318788.s008.docx]

Reviewers' comments:

- Have the authors made all data underlying the findings in their manuscript fully available?

The PLOS Data policy requires authors to make all data underlying the findings described in their manuscript fully available without restriction, with rare exception (please refer to the Data Availability Statement in the manuscript PDF file). The data should be provided as part of the manuscript or its supporting information, or deposited to a public repository. For example, in addition to summary statistics, the data points behind means, medians and variance measures should be available. If there are restrictions on publicly sharing data—e.g. participant privacy or use of data from a third party—those must be specified.

Response: We thank the reviewer for their comment. The data underlying our findings are fully available and comply with the PLOS Data Policy. Specifically, the following measures have been taken to ensure transparency and accessibility:

- We have included a detailed statement in the manuscript specifying that all data underlying the findings are available upon request by emailing the first author.
- The data points behind the means, medians, and variance measures mentioned in the manuscript have been preserved and are available for sharing. These data include the raw responses to the survey, stratified summary statistics, and supplementary analyses.
- Given the sensitive nature of the data and the need to protect participants' privacy, direct access to raw data is provided upon request. This approach aligns with ethical guidelines approved by the Institutional Review Board of the Faculty of Medicine, Alexandria University, Egypt (IRB number: 00012098).

If there are additional specifications or repositories preferred by the journal, we are willing to comply and upload the dataset accordingly.

- The study claims broad representation across various regions; however, clarifying the sampling method (e.g., were the samples proportionate to the healthcare workforce distribution?) would strengthen claims of cross-national comparison. Additionally, authors should specify why certain countries or regions may have lower representation could provide context for the sampling limitations.

Response: We thank the reviewer for this important observation. We acknowledge the need to clarify the sampling method and provide additional context regarding the representation of healthcare workers (HCWs) across regions. Below is our response:

- The study employed a convenience and snowball sampling approach to recruit HCWs from different countries. Collaborators from the Global Researcher Club (GRC), an international, voluntary, and non-profit research network, were tasked with recruiting participants from their respective regions. The sampling was not proportionate to the actual healthcare workforce distribution across countries or regions due to the limitations of available collaborators and logistical constraints.
- Although we aimed for broad representation, we acknowledge that the sample sizes from some regions (e.g., Western countries and certain parts of Eastern Asia and Latin America) are smaller compared to others like the Eastern Mediterranean and African regions. This disparity may reflect differences in the ease of access to HCWs, variations in collaborator networks, and the differing response rates among participants from these regions.
- We recognize that these sampling variations could introduce biases and limit the generalizability of our findings. However, we believe that the observed trends and associations remain valuable for highlighting regional disparities and guiding targeted interventions.
- We recommend that future studies consider employing proportionate sampling strategies or quota sampling methods based on the actual distribution of HCWs across regions. Additionally, using global health workforce statistics to guide recruitment efforts could improve representativeness.

We will include these clarifications in the revised manuscript under the "Methods" and "Limitations" sections to provide transparency and contextualize our findings.

- Since the authors report a correlation between mental health issues and vaccine hesitancy, additional context on how mental health was assessed or any screening tools used might be useful.

Response: We appreciate the reviewer’s valuable suggestion to provide additional context regarding the assessment of mental health issues, given its correlation with vaccine hesitancy in our study.

- Mental health was assessed in the study through self-reported responses to specific questions about the presence of mental health conditions, including stress, anxiety, sleep disorders, and obsessive-compulsive disorder (OCD). Participants were also asked if they had been diagnosed with schizophrenia or other psychiatric conditions. While these questions were based on participants' acknowledgment of existing conditions, either through personal awareness or prior diagnosis by a healthcare provider, they were not assessed using standardized tools.
- We recognize that the absence of validated screening tools, such as the Generalized Anxiety Disorder-7 (GAD-7) or Patient Health Questionnaire-9 (PHQ-9), limits the precision of our mental health measurements. We will include this limitation in the manuscript and emphasize the need for future studies to use validated instruments to enhance the robustness of findings related to mental health and vaccine hesitancy.
- We have added clarifications in the "Data Collection Tools" and "Discussion" sections of the manuscript, detailing how mental health issues were assessed and highlighting the limitations of the self-reported approach. These revisions aim to provide greater transparency and address the reviewer’s concern comprehensively.

Thank you for this insightful comment, which has strengthened our discussion of this important topic.

- The discussion might benefit from a more cautious interpretation of causality in observed relationships (e.g., family size, personal COVID-19 history) with vaccine confidence and fear. Since this is a cross-sectional study, it might be helpful to remind readers that causation cannot be firmly established.

Response: We fully acknowledge that the cross-sectional design of our study limits the ability to establish causation between variables such as family size or personal COVID-19 history and outcomes like vaccine confidence and fear. While we observed statistically significant associations, these should be interpreted as correlations rather than definitive causal relationships.

- To address this concern, we have revised the "Discussion" section to emphasize the observational nature of our findings. We have added the following clarification:

"It is important to note that the cross-sectional design of this study precludes any definitive conclusions about causality. The associations observed between variables such as family size or personal COVID-19 history and vaccine confidence or fear reflect correlations rather than causal relationships. Future longitudinal studies are necessary to explore these dynamics and determine causal pathways."

- This revision ensures that readers are reminded of the inherent limitations of the study design when interpreting the findings. Additionally, we will underscore the need for future studies employing longitudinal or experimental designs to further investigate these relationships.

Thank you for this valuable suggestion, which has enhanced the clarity and rigor of our discussion

- Although some limitations are implied, I recommend adding a dedicated limitations section discussing potential biases (e.g., self-reported data, convenience sampling) and limitations in the survey's cross-sectional design for a complete discussion.

Response: Done
