# Supplementary material for: Cross-national disparities in healthcare workers’ perceptions: Examining fear of infection and confidence in the received COVID-19 vaccines amid emerging variants
Source: PLoS One. 2025 Dec 12;20(12):e0318788. doi: 10.1371/journal.pone.0318788 (PMC12700404; doi:10.1371/journal.pone.0318788)
Supplement: Attachment — Submitted filename: RTR_auresp_3.docx [file pone.0318788.s009.docx]

Dear Professor Stephen

Thank you for giving us the opportunity to enhance the quality of this manuscript. We have carefully addressed typos, grammatical errors, and redundancies to ensure it meets the high standards of your prestigious journal.

Sincerely,

Ramy Mohamed Ghazy
