# Supplementary material for: Cross-national disparities in healthcare workers’ perceptions: Examining fear of infection and confidence in the received COVID-19 vaccines amid emerging variants
Source: PLoS One. 2025 Dec 12;20(12):e0318788. doi: 10.1371/journal.pone.0318788 (PMC12700404; doi:10.1371/journal.pone.0318788)
Supplement: Attachment — Submitted filename: RTR_MH-HS.docx [file pone.0318788.s010.docx]

**Dear Editorial Office,**

Thank you for reviewing the manuscript I submitted for PLOS ONE entitled (**Cross-National Disparities in Healthcare Workers' Perceptions: Examining Fear of Infection and Confidence in COVID-19 Vaccines amid Emerging Variants).** Your efforts in reviewing my manuscript are appreciated. Your comments have been instrumental in enhancing the quality of our research, and we have carefully considered them. We are pleased to present the revised version of our manuscript, incorporating the changes you suggested

We would like to inform you that our manuscript was initially accepted and sent to production. However, we made minor edits afterward to enhance the quality of our work. As a result, the manuscript underwent another round of review and received a minor revision decision. The editor apologized for this additional review round, which occurred solely because of our efforts to improve the manuscript’s quality

As regards your valuable comments on the following:

**Editor Comments:**

1- Title Revision: Please revise the title to include “…amid Emerging Variants” to better reflect the evolving pandemic context.

**Author response:** Thank you for your valuable suggestion. We agree that incorporating “amid Emerging Variants” enhances the relevance of the title within the evolving context of the COVID-19 pandemic. Accordingly, the revised title is: *“Cross-National Disparities in Healthcare Workers' Perceptions: Examining Fear of Infection and Confidence in COVID-19 Vaccines amid Emerging Variants.”*

2- Introduction Restructuring: The introduction is too long and lacks coherence. Some sections include information that is not directly relevant to the study. Please restructure this section to ensure a logical flow and conciseness.

**Author response:** Thank you for your valuable suggestion. It has been done as requested

3- Sample Selection Bias: The study relies on a convenience sample of HCWs with internet access, which may introduce selection bias, particularly in regions with limited connectivity. Please discuss this limitation and explain any measures taken to mitigate its impact.

**Author response:** Thank you for your valuable comments. The data was collected through an online anonymous survey and face-to-face distribution of hard copies of surveys to target participants during working hours. The online survey was uploaded to Google Forms, then distributed through social media platforms (Facebook and Twitter) and sent via email, WhatsApp, and Telegram. The printed hard copy of the study questionnaire was distributed to targeted HCWs to complete it to overcome any challenges with internet access.

Also, we recommended that future studies should consider employing proportionate or quota sampling strategies based on global health workforce distribution to improve representativeness.

4- Data Collection Tools: This section should be more concise. Move detailed survey questions to an appendix and minimize descriptions of previously validated scales, referring instead to original sources.

**Author response:** Thank you for your valuable comments. It was done as requested and the details on the supplementary file and all related and required information remain in the main manuscript

5- Questionnaire Language: Clarify whether the questionnaire was administered in English across all countries. If so, discuss potential limitations for non-native speakers.

**Author response:** Thank you for your valuable comments. the questionnaire was used in both Arabic and English versions

6- Results Section Revision: Given the large number of tables and figures, consider moving sociodemographic characteristics to a supplementary file. Additionally, refine the narrative to avoid excessive repetition of data already presented in the tables.

**Author response:** Thank you for your suggestion. However, we believe it is important to keep the sociodemographic characteristics table in the main manuscript for the following reasons:

- It describes our sample in detail and demonstrates its balance across key demographics such as age, gender, and profession.
- These characteristics are directly related to the main outcomes (fear and vaccine confidence) and help the reader interpret our findings more clearly.
- We also wanted to ensure transparency, especially in a multi-country study, where understanding the sample makeup is essential.

Regarding the narrative, we were careful to highlight only the most important findings and avoid repeating data already shown in the tables.

7- Sample Representativeness: The rationale for selecting specific countries is unclear. Please clarify why these countries were chosen and assess whether the sample represents the broader HCW population in each country based on demographics such as gender, age, and occupation.

**Author response:** Thank you for this insightful comment. We appreciate the opportunity to clarify the rationale for our country selection and address the representativeness of our sample.

The countries included in the study were selected based on the feasibility of data collection through our existing network of collaborators in the Global Researcher Club (GRC), an international, voluntary, and non-profit scientific research network. Our aim was to achieve a diverse sample across multiple WHO regions, including the Eastern Mediterranean Region (EMR), African Region (AFR), Western Region, and other global areas such as Asia and Latin America. Collaborators from these countries had prior experience with survey-based research and were able to facilitate recruitment and translation where necessary.

We acknowledge that this convenience and snowball sampling approach may limit the representativeness of the sample. To address this concern, we have now added a section to the Discussion highlighting this limitation and recommending that future studies use quota or stratified sampling to ensure representativeness in terms of gender, age, and professional categories across countries.

8- Multivariate Analysis: The analysis does not appear to adjust for country-level differences, which may be a confounding factor. Given the diversity of the countries studied, it is crucial to account for these variations in attitudes toward vaccination. Please consider adjusting for country in the analysis.

**Author response:** Thank you for this important point. We agree that cultural and socioeconomic differences across countries may influence fear and vaccine confidence. However, our goal was to explore generalizable trends across regions, and our multivariate analysis adjusted for world region (e.g., EMR, AFR, Western, Other), which we used as a proxy for broader contextual differences.

While adjusting for individual countries was limited by unequal sample sizes across countries, this may have reduced model stability or introduced bias. We acknowledge this as a limitation and have added a statement in the Discussion section recommending that future studies use country-level adjustment or stratified analyses where sample sizes allow.

9- Discussion Section Restructuring: The discussion is lengthy and difficult to follow. Structuring it into clear subsections will improve readability and enhance the clarity of your key findings.

**Author response:** Thank you for your valuable feedback. We appreciate your suggestion regarding the structure of the Discussion section. In response, we have carefully revised and reorganized the section into clearly defined subsections, each addressing a specific aspect of our findings.

Additionally, there is an interesting contrast between your findings and existing literature on vaccine hesitancy in the general population. Studies such as Dumitra et al. identify vaccine refusal primarily due to perceived lack of necessity or belief in natural immunity, while your study suggests that prior COVID-19 infection and vaccine side effects were associated with lower vaccine confidence among HCWs. This difference may be due to professional exposure, firsthand experience with COVID-19 severity, and evolving attitudes across different pandemic waves. Furthermore, studies like Savulescu et al. suggest that declining disease severity and the relatively short-lived protection of boosters may have influenced HCWs’ vaccine confidence. Incorporating this perspective into the discussion will strengthen your argument.

**Author response:** Thank you for this thoughtful and insightful comment. We agree that contrasting our findings with existing literature on vaccine hesitancy in the general population adds important context to our study. We have now integrated this perspective into the revised Discussion section, specifically under the subsection “Interplay Between Fear and Vaccine Confidence.” We also referenced studies by Dumitra et al. and Savulescu et al. to highlight how the professional experiences of HCWs may shape their confidence in COVID-19 vaccines differently from the general population. This addition helps contextualize our findings within the broader literature and enhances the strength of our interpretation.

**Reviewers' comments:**

Reviewer #3:

1- Several studies addressing vaccine hesitancy or acceptance in lay-people are documenting a more complex picture than just fear/extreme fear. A recent paper [Dumitra GG et al - Segmenting attitudes toward vaccination – behavioural insights into influenza vaccination refusal in Romania. GERMS. 2024;14(4):362-374. doi: 10.18683/germs.2024.1446] documented 3 distinct latent-class groups of people regarding vaccine and disease perception. Highest refusal was present in people that perceived lack of necessity and belief in natural immunity. A smaller degree of refusal was documented in people with past negative experiences with vaccines or distrust in vaccines. Can you comment about these findings compared with your statement in page 25 *"personal history of COVID-19 infection, and experiencing side effects of COVID vaccination were associated with lower vaccine confidence"* after stating in page 24 *"Research investigating vaccine confidence in HCWs with a previous history of COVID-19 infection also yields mixed results, with some studies reporting high confidence [75], while others suggest low confidence [76]. It is worth emphasizing that the firsthand experience of the disease's severity and its impact play a crucial role in shaping confidence toward vaccination"*

**Author response:** Thank you for this insightful observation and for highlighting the study by Dumitra et al., which enriches the discussion on the heterogeneity of vaccine hesitancy. We fully agree that vaccine attitudes extend beyond simple fear and are shaped by multiple psychological, experiential, and cognitive dimensions.

To address this, we have now expanded the relevant section of the Discussion to better contrast our findings among HCWs with the behavioural segmentation observed in the general population. Specifically, we highlight that while Dumitra et al. identified a lack of perceived necessity and belief in natural immunity as key predictors of hesitancy in the lay public, our study among HCWs revealed that prior COVID-19 infection and negative post-vaccination experiences were associated with decreased confidence. This contrast likely reflects differences in baseline health literacy, exposure to clinical realities, and risk assessment frameworks.

2- These seem to document different perception models from HCW in your study and general population, probably because of major differences in disease perception [did you ask in your study if responding HCW were working in a unit where COVID-19 patients were isolated/treated?].

**Author response:** Thank you for this thoughtful comment. You are correct in noting that disease perception models likely differ between HCWs and the general population due to differences in risk exposure and professional experience. While our survey captured detailed sociodemographic and health information, including history of COVID-19 infection and vaccine side effects—it did not specifically ask whether HCWS were assigned to COVID-19 treatment or isolation units. We acknowledge this as a limitation in the study and have added a statement to the Discussion section to reflect this. We also suggest that future research incorporate clinical role or unit assignment as a potential modifier of vaccine confidence and fear.

3- Experiencing first-hand disease severity decrease during different waves of pandemic (after Delta wave all subsequent Omicron lineages were less and less devastating), several groups of HCW decreased in fear and potentially developed a more relaxed behaviour and less adoption of recurrent boosters. Some sense of "futility" of boosting was inflicted by a certain perception bias, when confronted with the relative short period of protection gained by boosting as documented in an European study performed in 10 countries and 19 hospitals (Savulescu C, et al. Incidence of SARS-CoV-2 Infection Among European Healthcare Workers and Effectiveness of the First Booster COVID-19 Vaccine, VEBIS HCW Observational Cohort Study, May 2021–May 2023. Vaccines. 2024; 12(11):1295. <https://doi.org/10.3390/vaccines12111295>).

**Author response:** We appreciate the reviewer’s insightful comment highlighting the evolving dynamics of disease severity perception among HCWs. Indeed, as the pandemic progressed and Omicron lineages replaced more severe variants like Delta, HCWs may have adjusted their risk assessments, leading to a decline in fear and a perceived diminishing value in repeated booster vaccinations. We have now incorporated this point into the Discussion section and cited the European VEBIS HCW Cohort Study by Savulescu et al. (2024), which supports the observation of declining perceived utility of boosters due to their short-lived protective effect.

Reviewer #4:

1- Title: Please revise to include the phrase “…amid Emerging Variants”

**Author response:** Thank you for your valuable suggestion. We agree that incorporating “amid Emerging Variants” enhances the relevance of the title within the evolving context of the COVID-19 pandemic. Accordingly, the revised title is: *“Cross-National Disparities in Healthcare Workers' Perceptions: Examining Fear of Infection and Confidence in COVID-19 Vaccines amid Emerging Variants.”*

2- Introduction: The section is too long and needs to be restructured. It includes a significant amount of information that is not directly relevant to the study. Additionally, there is a lack of coherence between successive paragraphs, which seem to have been written independently. The introduction should have a clear and logical flow, which is currently missing. Please revise.

**Author response:** Thank you for this insight. It has been reconstructed for better coherence.

3- Sample Selection Bias: The study relies on a convenience sample, including only healthcare professionals with internet access. Could this introduce a significant selection bias, particularly in countries where internet access may be limited? How was this issue addressed? Was any measure taken to mitigate its impact

**Author response:** Thank you for your valuable comments. The data was collected through an online anonymous survey and face-to-face distribution of hard copies of surveys to target participants during working hours. The online survey was uploaded to Google Forms, then distributed through social media platforms (Facebook and Twitter) and sent via email, WhatsApp, and Telegram. The printed hard copy of the study questionnaire was distributed to targeted HCWs to complete it to overcome any challenges with internet access.

Also, we recommended that future studies should consider employing proportionate or quota sampling strategies based on global health workforce distribution to improve representativeness.

4- Data Collection Tools section: This section should be more concise, with detailed information on survey questions moved to an appendix. Additionally, since previously validated scales were used, their descriptions should be minimized, referring readers to the original publications for further details.

**Author response:** Thank you for your valuable comments. It was done as requested and the details on the supplementary file and all related and required information remain in the main manuscript

Additionally, there is an interesting contrast between your findings and existing literature on vaccine hesitancy in the general population. Studies such as Dumitra et al. identify vaccine refusal primarily due to perceived lack of necessity or belief in natural immunity, while your study suggests that prior COVID-19 infection and vaccine side effects were associated with lower vaccine confidence among HCWs. This difference may be due to professional exposure, firsthand experience with COVID-19 severity, and evolving attitudes across different pandemic waves. Furthermore, studies like Savulescu et al. suggest that declining disease severity and the relatively short-lived protection of boosters may have influenced HCWs’ vaccine confidence. Incorporating this perspective into the discussion will strengthen your argument.

**Author response:** Thank you for this thoughtful and insightful comment. We agree that contrasting our findings with existing literature on vaccine hesitancy in the general population adds important context to our study. We have now integrated this perspective into the revised Discussion section, specifically under the subsection “Interplay Between Fear and Vaccine Confidence.” We also referenced studies by Dumitra et al. and Savulescu et al. to highlight how the professional experiences of HCWs may shape their confidence in COVID-19 vaccines differently from the general population. This addition helps contextualize our findings within the broader literature and enhances the strength of our interpretation.

5- Language of the Questionnaire: was the questionnaire administered in English in all countries? If so, could this be a limitation for participants in certain regions?

**Author response:** Thank you for your valuable comments. the questionnaire was used in both Arabic and English versions

6- Results Section: Given the large number of tables and figures, I suggest moving the initial tables on sociodemographic characteristics to a supplementary file. Moreover, the narrative description is overly detailed, often repeating information already provided in the tables. Please revise.

**Author response:** Thank you for your suggestion. However, we believe it is important to keep the sociodemographic characteristics table in the main manuscript for the following reasons:

- It describes our sample in detail and demonstrates its balance across key demographics such as age, gender, and profession.
- These characteristics are directly related to the main outcomes (fear and vaccine confidence) and help the reader interpret our findings more clearly.
- We also wanted to ensure transparency, especially in a multi-country study, where understanding the sample makeup is essential.

Regarding the narrative, we were careful to highlight only the most important findings and avoid repeating data already shown in the tables.

7. Sample Representativeness: While the study appears methodologically well performed, I have concerns about the representativeness of the sample. First, the rationale for grouping these specific countries together is unclear. Second, to what extent do the selected healthcare professionals represent the broader population of healthcare workers in each country? Was any assessment of representativeness conducted? For example, does the sample reflect national distributions in terms of gender, age group, and occupation?

**Author response:** Thank you for this insightful comment. We appreciate the opportunity to clarify the rationale for our country selection and address the representativeness of our sample.

The countries included in the study were selected based on the feasibility of data collection through our existing network of collaborators in the Global Researcher Club (GRC), an international, voluntary, and non-profit scientific research network. Our aim was to achieve a diverse sample across multiple WHO regions, including the Eastern Mediterranean Region (EMR), African Region (AFR), Western Region, and other global areas such as Asia and Latin America. Collaborators from these countries had prior experience with survey-based research and were able to facilitate recruitment and translation where necessary.

We acknowledge that this convenience and snowball sampling approach may limit the representativeness of the sample. To address this concern, we have now added a section to the Discussion highlighting this limitation and recommending that future studies use quota or stratified sampling to ensure representativeness in terms of gender, age, and professional categories across countries.

8. Multivariate Analysis: The analysis does not appear to adjust for country. Given the diversity of the countries studied, do you not consider this a potential confounding factor? Beliefs and attitudes toward vaccination are likely influenced by the socioeconomic and cultural contexts of each country, making it essential to account for these differences in the analysis. Please consider this suggestion.

**Author response:** Thank you for this important point. We agree that cultural and socioeconomic differences across countries may influence fear and vaccine confidence. However, our goal was to explore generalizable trends across regions, and our multivariate analysis adjusted for world region (e.g., EMR, AFR, Western, Other), which we used as a proxy for broader contextual differences.

While adjusting for individual countries would have added nuance, doing so was limited by unequal sample sizes across countries, which may have reduced model stability or introduced bias. We acknowledge this as a limitation and have added a statement in the Discussion section recommending that future studies use country-level adjustment or stratified analyses where sample sizes allow.

9. Discussion Section: The discussion lacks structure and is difficult to follow. Given its length, I recommend restructuring it into subsections to improve readability and comprehension.

**Author response:** Thank you for your valuable feedback. We appreciate your suggestion regarding the structure of the Discussion section. In response, we have carefully revised and reorganized the section into clearly defined subsections, each addressing a specific aspect of our findings.
