# Supplementary material for: Cross-national disparities in healthcare workers’ perceptions: Examining fear of infection and confidence in the received COVID-19 vaccines amid emerging variants
Source: PLoS One. 2025 Dec 12;20(12):e0318788. doi: 10.1371/journal.pone.0318788 (PMC12700404; doi:10.1371/journal.pone.0318788)
Supplement: Attachment — Submitted filename: RTR_auresp_5.docx [file pone.0318788.s012.docx]

Dear Reviewer Team,

Thank you very much for your time and feedback. We go over all the comments and provide our responses.

**Additional Editor Comments:**

The aim of this research is timely and relevant, particularly in the context of ongoing global discussions surrounding vaccine hesitancy and the evolving dynamics of the COVID-19 pandemic. While the cross-sectional design and use of a simple questionnaire limit the depth of scientific inquiry, the inclusion of participants from multiple countries adds value by offering broad, generalized insights that could inform future research and public health policy. However, several critical revisions are necessary to strengthen the manuscript.

- First, clarify abbreviations such as "VH" upon first use and specify what is included under "others" in the place of residence category.

Response: Thank you for your comment. We have revised the manuscript to clarify what is included under the “others” category for place of residence. Specifically, “others” refers to healthcare workers who reported non-standard or unlisted living areas such as refugee camps, temporary housing, or remote settlements that do not fall under the predefined categories of urban/city, rural/village, or desert/mountain.

- Please also ensure that all figures are correctly displayed and embedded in the manuscript.

Response: Done

- Consider whether it would be beneficial to indicate the version of the vaccine (monovalent or bivalent) to align with variant-specific developments.

Response: Thank you for this insightful suggestion. Unfortunately, the survey did not collect information on the specific version of the COVID-19 vaccine (monovalent vs. bivalent) received by participants. At the time of data collection, most vaccinations administered in the participating regions were from the initial rollout phases, and bivalent vaccines were not yet widely available. Therefore, we were unable to differentiate vaccine versions in our analysis. We have added a note to the Limitations section to reflect this point.

- To improve the clarity and conciseness of the Results section, we recommend removing Tables 1 and 2, as their content is already described in the text, and consider including Supplementary Table 2, which appears to be missing. Addressing these points will enhance the manuscript’s overall clarity, relevance, and impact for a broad audience.

Response: Done

**Reviewer #3:**

Dear colleagues, thank you for the persistent effort made in improvement of your research especially in the Discussion section that was significantly improved by inserting clarifications in sections like "Fear of Emerging COVID-19 Variants Among HCWs", "Confidence in the received COVID-19 Vaccines" or "Interplay between fear of new SARS-Cov-2 variant infection and confidence in the received vaccine". Also, valuable improvements have been implemented in Study Limitations section. Congratulations on your detailed revision of this paper.

Response: Thank you very much for your feedback and advice

**Reviewer #5:**

Thanks to all authors for their competitive work, please find some comments below:

1- Introduction: A study on 2,336 HWCs reported an increasing rate of severe COVID-19 fear from 9% to 15% please give details how you concluded its increase, is it a longitudinal study?

Response:
We appreciate the reviewer’s thoughtful observation. The referenced study by Moretti et al. (2022) is indeed a prospective cohort study, conducted at a single center, which followed healthcare workers over time. The reported increase in severe COVID-19 fear from 9% to 15% reflects a temporal change within the same cohort, confirming that the study had a longitudinal design.

2- Methods: You mentioned (A nationwide); however, this study has a global outreach, what nation did you mean?

Response:
Thank you for highlighting this inconsistency. You are correct, the study involved participants from multiple countries and regions, and the term “nationwide” inaccurately implies it was restricted to a single country. We have revised the wording in the Methods section to reflect the global scope of the survey.

3- Regarding age distribution, 86 was the maximum, this age for healthcare worker is a bit weird.

Response:
Thank you for this insightful comment. We agree that 86 years may seem atypical for an actively practicing healthcare worker. However, our study included a broad definition of healthcare workers, encompassing not only clinical staff but also retired professionals still engaged in part-time roles or consultancy, academic staff, and volunteers involved in healthcare settings. In some countries, particularly where healthcare infrastructure is stretched, retired professionals or older individuals may continue contributing to the healthcare system in advisory, supervisory, or training capacities.

4- In conclusion, HCWs exhibited notable fear of SARS-CoV-2 new variants with low confidence. Better to add infection to be notable fear of infection with SARS-CoV-2 new variants with low confidence

Response: Done

5- Sample size calculation: We multiplied by 4 to compensate for stratification (Western region, Arab region, African region, and Others). I would advise you to add the total sample size

Response: Done

6- Kindly note that WHO classifies variants as variants of concern, variants of interest and variants under monitoring. CDC classifies variants as variants of concern, variants of interest, and variants of high consequence. Nomenclature by WHO is a bit more recent.

Response: In this study, we used the term “emerging variants” to refer broadly to newly identified or recently spreading SARS-CoV-2 variants, without assigning them to specific official classifications.

7- Do you add the version of vaccine Monovalent / bivalent to coincide with the updated form of variants?

Response: Thank you for this insightful suggestion. Unfortunately, the survey did not collect information on the specific version of the COVID-19 vaccine (monovalent vs. bivalent) received by participants. At the time of data collection, most vaccinations administered in the participating regions were from the initial rollout phases, and bivalent vaccines were not yet widely available. Therefore, we were unable to differentiate vaccine versions in our analysis. We have added a note to the Limitations section to reflect this point.

8- Please clarify what VH is the first time its mentioned in the text.

Response: Done

9- Please take care that figures are not displayed in the manuscript provided to me for review, you must ensure their inclusion.

Response: Done

10- Place of residence included others, please define what the others are?

Response: Thank you for your comment. We have revised the manuscript to clarify what is included under the “others” category for place of residence. Specifically, “others” refers to healthcare workers who reported non-standard or unlisted living areas such as refugee camps, temporary housing, or remote settlements that do not fall under the predefined categories of urban/city, rural/village, or desert/mountain.

11- Discussion: Key brain regions, such as the amygdala, insula, and prefrontal cortex, play important roles in processing fear, risk perception, and decision making. What does this statement add to the discussion?

Response:
Thank you for your insightful comment. We agree that the original statement required further contextualization. We have revised the paragraph to directly relate the neurocognitive basis of fear and risk perception to the observed extreme fear among HCWs. This addition helps to explain the psychological underpinnings behind fear responses to emerging variants and how such fear may influence behavior and attitudes toward vaccination.

**Reviewer #6:**

The aim of this research is pertinent topic for examination at this phase of pandemic and with common concerns about the pharmaceutical intervention. The cross-sectional study design, with broad questions in simple questionnaire survey, mean that depth is lacking for true scientific inquiry (thus, "partly" for Review Question #2), but the inclusion of multiple countries means that these generalized findings may help understanding for non-specific audience of readers. The claims could be useful, even if refuted by more rigorous design, for further medical research and informing public health policy.

With these strengths, there are some suggestions (in response to Question #1) made by previous reviewers which could vastly improve the study manuscript. I agree that the Results sections has too many tables with the same information already written in the text.

- My suggestion is to delete Tables 1 and 2 to reduce the size of this section and possibly include the Supplementary Table #2 (unavailable in the current manuscript).

Response: Done

- Major omissions in documents will require fix for the final submission (in response to Question #4): Figure 1 & 2 are missing from Pages 24 & 27 in the version I received; and there is no "Supplementary Table 2" available. I was unable to review these inserts of results.

Response: Done

As noted in previous reviews and still not resolved satisfyingly, the wording in the manuscript is imprecise and also the flow of introduction & discussion are too general and off-topic ("no" on Question #5) seemingly like an early draft. I have some specific improvements here:

- Page 1: Short title: Misspelling "SARS-Cov2"

Done

- Page 1: Abstract conclusion: Revise to "In conclusion, HCWs exhibited notable fear of emerging SARS-CoV2 variants and there is low level of confidence in COVID-19 vaccination"

Done

- Page 16: In stead of "vaccine effectiveness concerns surfaced", there were new questions about "duration of protection" and "effectiveness against new variants" questions post-Omicron variant emergence

Done

- "SARS-Cov-2 vaccine approval" should be written "COVID-19 vaccine approval,".. Moreover, there are concerns over "how immunity from vaccines might influence viral evolution of spike protein"

Done

- Page 18: Use "of either sex" because you can't have a study participant who is both sexes at once

Done

- Page 23: Hypertension is chronic "condition" not disease. Use "the most reported health problems"

Done

- Page 28: Table 5 Row title use "Area of practice in healthcare work" deleting Field of study to support statement in discussion on Page 32 "Working as a physician, dentist, pharmacist, or physiotherapist was associated with lower levels of fear."

Done

- Page 31 "Neural substrates" is off topic and should be removed. Focus discussion on study related measurements, like which HCW had increased fear, or Discussion documented number of deaths in surveyed countries correlate with fear?

Clarified

- Again, a general discussion about marital happiness is out of focus on COVID-19 fear. What about illness support through marriage

Response:

Thank you for this insightful comment. We agree that a general discussion of marital happiness may diverge from the core focus of our study. In response, we have revised the discussion to emphasize how marital relationships may offer emotional and practical support during illness, which could help mitigate fear associated with infectious disease outbreaks.

- Page 32 Discussion missed a large issue of interest, on if Confidence is related according to vaccine received?

Response:
Thank you for this important observation. We acknowledge that the discussion did not explicitly address the potential association between the type of vaccine received and healthcare workers’ confidence in COVID-19 vaccination. While our study collected data on vaccine types, our primary analysis did not stratify confidence levels by specific vaccine brands. This can be an area for further investigation.

- Page 34 What about high non-response rate? Is the missing population a limitation in your findings?

Response:

Thank you for raising this important point. We acknowledge that our study did not formally assess or report the response rate, as the survey was disseminated through online platforms using a snowball sampling technique, and the denominator of those who received the survey is unknown. Therefore, we could not estimate the non-response rate accurately.

- Page 34 Length of time (2 years +) since vaccines introduced should be discussed as aspect of study finding on confidence of vaccine (as well as fear of new variants); Furthermore, have better treatments become available during the time since pandemic to ease fears of death and severe illness from new COvID-19 infection

Done
