# Supplementary material for: Cross-national disparities in healthcare workers’ perceptions: Examining fear of infection and confidence in the received COVID-19 vaccines amid emerging variants
Source: PLoS One. 2025 Dec 12;20(12):e0318788. doi: 10.1371/journal.pone.0318788 (PMC12700404; doi:10.1371/journal.pone.0318788)
Supplement: Attachment — Submitted filename: Comments COVID19 FEAR - REVIEWER5.doc [file pone.0318788.s011.doc]

3 October 2025

Comments for COVID-19 Fear manuscript - REVIEWER 5

Overall: Accept. The author has revised the manuscript and made substantial changes based on the reviewer feedback. The document is now acceptable for publication.

Remaining Correction:

Page 13 of Results (23 of Document: “Approximately 30%” or “Exactly 30.4%” not Exact to start sentence
